# Novel Pathway of Adipogenesis through Cross-Talk between Adipose Tissue Macrophages, Adipose Stem Cells and Adipocytes: Evidence of Cell Plasticity

**DOI:** 10.1371/journal.pone.0017834

**Published:** 2011-03-31

**Authors:** Gregorio Chazenbalk, Cristina Bertolotto, Saleh Heneidi, Medet Jumabay, Bradley Trivax, Joel Aronowitz, Kotaro Yoshimura, Charles F. Simmons, Daniel A. Dumesic, Ricardo Azziz

**Affiliations:** 1 Department of Obstetrics and Gynecology, David Geffen School of Medicine at University of California Los Angeles, Los Angeles, California, United States of America; 2 Department of Pediatrics, Division of Neonatology, Cedars-Sinai Medical Center, Los Angeles, California, United States of America; 3 Department of Neonatology, David Geffen School of Medicine at University of California Los Angeles, Los Angeles, California, United States of America; 4 Department of Obstetrics and Gynecology, Medical College of Georgia, Augusta, Georgia, United States of America; 5 Department of Cardiology, David Geffen School of Medicine at University of California Los Angeles, Los Angeles, California, United States of America; 6 Division of Plastic Surgery, Department of Surgery, Cedars-Sinai Medical Center, Los Angeles, California, United States of America; 7 University of Southern California, Los Angeles, California, United States of America; 8 Department of Plastic Surgery, University of Tokyo, Tokyo, Japan; Cedars-Sinai Medical Center, United States of America

## Abstract

**Introduction:**

Previous studies highlight a complex relationship between lineage and phenotype for adipose tissue macrophages (ATMs), adipose stem cells (ASCs), and adipocytes, suggesting a high degree of plasticity of these cells. In the present study, using a novel co-culture system, we further characterized the interaction between ATMs, ASCs and adipocytes.

**Research Design and Methods:**

Human adipocytes and the stromal vascular fraction containing ATMs and ASCs were isolated from human adipose tissue and co-cultured for 24 hours. FACS was used to characterize ATMs and ASCs before and after co-culture. Preadipocytes generated after co-culture were characterized by immunostaining for DLK (preadipocytes), CD14 and CD68 (ATMs), CD34 (ASCs), and Nile Red staining for lipid drops. qRT-PCR was used to quantify adipogenic markers such as C/EBPα and PPARγ. A novel fluorescent nanobead lineage tracing method was utilized before co-culture where fluorescent nanobeads were internalized by CD68 (+) ATMs.

**Results:**

Co-culture of adipocytes with ATMs and ASCs increased the formation of new preadipocytes, thereby increasing lipid accumulation and C/EBPα and PPARγ gene expression. Preadipocytes originating after co-culture were positive for markers of preadipocytes, ATMs and ASCs. Moreover, fluorescent nanobeads were internalized by ATMs before co-culture and the new preadipocytes formed after co-culture also contained fluorescent nanobeads, suggesting that new preadipocytes originated in part from ATMs. The formation of CD34(+)/CD68(+)/DLK (+) cell spheres supported the interaction of ATMs, ASCs and preadipocytes.

**Conclusions:**

Cross-talk between adipocytes, ATMs and ASCs promotes preadipocyte formation. The regulation of this novel adipogenic pathway involves differentiation of ATMs to preadipocytes. The presence of CD34(+)/CD68(+)/DLK(+) cells grouped in spheres suggest that paracrine interactions between these cell types plays an important role in the generation and proliferation of new preadipocytes. This phenomenon may reflect the in vivo plasticity of adipose tissue in which ATMs play an additional role during inflammation and other disease states. Understanding this novel pathway could influence adipogenesis, leading to new treatments for obesity, inflammation, and type 2 diabetes.

## Introduction

Obesity is a major contributor to chronic disease and disability, including type 2 diabetes [1]. The role of adipose tissue in obesity was thought to be a passive one, however, today it is understood that adipocytes play a much more active role in metabolism, including interactions with the immune system through inflammatory mediators and signaling molecules [2]–[3]. This inflammatory response appears to be critical in the development of obesity and later, insulin resistance [4]. In addition, adipose tissue macrophages (ATMs) and cytokines are able to keep preadipocytes in quiescent stages, and an imbalance in this mechanism could exacerbate the development of obesity and insulin resistance [4]–[5].

Macrophage expression of adipokine receptors for both leptin and adiponectin suggests that adipocytes may also modulate macrophage function [6]–[7]. *In vitro* co-culture of differentiated 3T3-L1 adipocytes and RAW 264 macrophages results in significant upregulation of proinflammatory cytokines and downregulation of anti-inflammatory cytokines in the macrophages [8]. Furthermore, the interaction of 3T3-L1 adipocytes with mouse peritoneal macrophages mediates the production of factors from macrophages that influence insulin sensitivity in adipocytes [9]. Recent studies demonstrated that co-culture of 3T3-L1 adipocytes with C2D macrophages inhibits insulin mediated glucose transport, adipocyte differentiation and diminishes macrophage function [10]. Understanding the range of interactions between adipocytes and macrophages may elucidate mechanisms underlying the etiology of excess adiposity and obesity.

Adipose tissue is not only composed of adipocytes, macrophages, and vascular tissue, but it also contains adult adipose stem cells (ASCs), that can be found in the adipose tissue derived stromal cell fraction [11]–[15]. These mesenchymal stem cells first become preadipocytes, which then can differentiate to adipocytes [11]–[14], [16]–[17]. The presence of CD68 (+)/CD34 (+) cells in adipose tissue has been recently described in db/db mice. The authors described a possible role of these cells in adipogenesis and angiogenesis [15].

ASCs can differentiate along adipocyte, osteoblast, chondrocyte, and other mesenchymal cell lineages in a manner similar to that of multipotent stromal cells derived from bone marrow [16]–[18]. It is generally accepted that mature adipocytes do not regularly undergo mitosis, and thus, an increase in adipocytes usually reflects a differentiation of preadipocytes [13]–[14]. However, several studies indicate that mature adipocytes could also have proliferative activity [19]–[20]. Recent studies suggest adipocytes can dedifferentiate to preadipocytes [21] and can even differentiate to a multipotent cell population [22], [23]. Of note, adipocyte precursors and preadipocytes have also been recently observed to rapidly and efficiently differentiate into typical macrophages [24], [25] demonstrating significant plasticity of these cells. Nevertheless, the role of ATMs in adipose tissue biology is still controversial. In the present study, we demonstrated that co-culture of adipocytes with ATMs and ASCs results in the robust proliferation of preadipocytes. In addition, these new preadipocytes can rapidly turn into adipocytes. ATMs can differentiate to preadipocytes as determined by lineage tracing. This novel pathway of generation of new preadipocytes/adipocytes also involved the formation of ATM/ASC/preadipocyte cell spheres. Thus, this paracrine cross-talk may reflect the *in vivo* plasticity of adipose tissue.

## Materials and Methods

### Subjects

Human adipose tissue samples were obtained from female patients undergoing abdominoplasty. All patients were premenopausal, non-diabetic, and none had been on any hormonal treatment, including oral contraceptives. Abdominal adipose tissue was excised and placed in buffer (12.5 mM Hepes Krebs-Ringer medium, 4% BSA, 2 mM pyruvate, pH 7.4**,** at room temperature (RT). A small amount of tissue was fixed in 4% PBS buffered paraformaldehyde and subjected to immunofluorescence, and the remainder was used for adipocyte and macrophage isolation. All studies were approved by the Cedars-Sinai Medical Center and the University of California Los Angeles, Institutional Review Board.

### Isolation of adipocytes and ATM/ASC fraction from human adipose tissue

Human adipose tissue was finely minced and treated with collagenase (Worthington Biochemical Corp., Lakewood, NJ) for 60 minutes at 37°C, in the transport buffer. The cell suspension was then filtered through a pre-moistened 150-micron nylon mesh (Small Parts Inc., Miami Lakes, FL) and centrifuged for 2 min at 50 xg at RT. The upper phase (floating adipocytes) was separated from lower phase. Adipocytes were washed twice and diluted in adipocyte culture medium (DMEM, 1% BSA, 3% FCS, 100 U/ml Penicillin, 100 µg/ml Streptomycin). The lower phase was subjected to centrifugation for 5 minutes at 500 xg. The cell pellet (ATM/ASC fraction) was resuspended in PBS and a Ficoll density gradient [26] was used to remove lymphocytes (Lymphoprep, Greiner Bio-one, Longwood). The interface containing the ATM/ASC fraction was removed and washed with 5 ml of PBS at RT. After a final centrifugation for 5 minutes at 500 xg, the cell pellet was resuspended in regular culture medium (RPMI medium supplemented with 10% FCS, 100 U/ml penicillin, 100 µg/ml streptomycin, 2 mM L- glutamine, 1% NEAA, 1% sodium pyruvate, and 10 ng/ml GM-CSF).

### Co-culture of adipocytes and the ATM/ASC fraction

**Figure 1:**
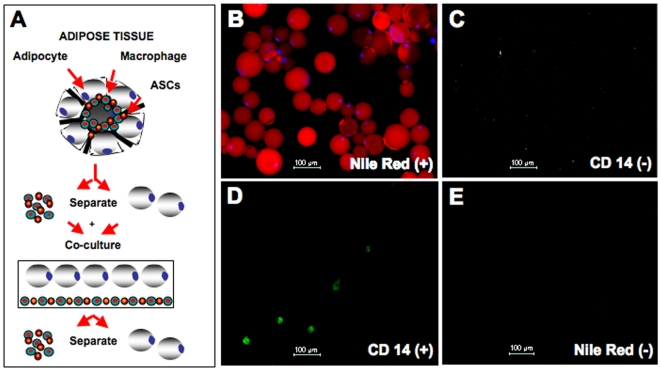
Scheme of co-culture between adipocytes, and ATMs/ASCs isolated from adipose tissue. Characterization of the isolated adipocytes and ATMs present in the ATM/ASC fraction. Isolated adipocytes and ATMs/ASCs were cultured separately for 24 hours to allow the cells to reach equilibrium and then were co-cultured for an additional 24 hours. Finally, the cells were separated and cultured for another 24 hours or 48 hours. (**A**) At the end of these periods, the culture media and the cells were subjected to different studies. (**B**) Nile Red co-labeled with DAPI (light blue) indicates the presence of mature adipocytes (x100). (**C**) Immunofluorescence (40X) of adipocytes cultured for 24 hours, cells are CD14 (−). (**D**) ATMs/ASCs after 3 days in culture, labeled for CD14 (200X), (**E**) these cells are Nile Red (-).

Cells from the ATM/ASC fraction (10^6^/ml) and isolated adipocytes (10^6^/ml) were allowed to equilibrate separately overnight in their respective cell culture media. Coverslips were placed in each well for immunofluorescence studies. Twenty-four hours later, resuspended adipocytes were added to the wells containing the macrophage fraction at an approximate 1∶1 ratio. The pooled cells were co-cultured for 24 hours at 37°C in 5% CO_2_. At the end of the incubation period the adipocytes were resuspended and transferred by pipette and placed in another well containing 1 ml of adipocyte culture medium (see above); the remaining ATM/ASC fraction was washed five times with 1 ml PBS, and cultured for an additional two or seven days in fresh regular medium (see above) or adipogenic medium (ZenBio, Research Triangle Park, NC) (**Fig. 1A** **)**. Coverslips were then collected for immunofluorescence (see below**).** Adipocytes and ATM/ASC fractions were also separately cultured under the same conditions described above.

### Flow cytometry analysis

The ATM/ASC fraction with and without co-culture with adipocytes was detached from dish using 1 mM EDTA/EGTA in PBS. After two washes in 2% inactivate FCS/0.05% sodium Azide/PBS, cells were resuspended in the same buffer and incubated at 4°C for 1 hour in the presence or absence of primary antibodies against i) CD14, a macrophage/monocyte specific marker (Biosource, Camarillo, CA); ii) CD34, an adult hematopoietic stem cell marker (Zymed, San Francisco, CA), and iii) DLK, a marker of preadipocytes and newly formed adipocytes (Santa Cruz, CA). Cells were then washed two times with same buffer described above and incubated with the corresponding secondary antibodies for 30 minutes at RT. After two consecutive washes in buffer, cells were resuspended in 100 µl of 2% inactivate FCS/0.05% Sodium Azide/PBS. Analysis of number and type of cells were performed using a FACS Calibur flow cytometer and cEllQuest Pro software.

### Differentiation of preadipocytes to adipocytes

To further characterize the adipogenesis capacity of the new preadipocytes generated after co-culture, confluent cells from ATM/ASC fractions with and without co-culture were kept for an additional seven days in Regular medium (see above) or in adipogenic medium (see above).

### Quantitative Real Time PCR (q-RTPCR)

Total RNA was isolated using RNeasy kits (Qiagen, Hilden, Germany). First strand cDNA was synthesized using RT2 first strand Master Mix (SABioscience, Frederick, MD) according to the manufacturer's instructions. The primers for C/EBPα and PPARγ for real-time PCR were obtained from Applied Biosystems (Foster City, CA). qRT-PCR was performed using Taqman® Gene Expression kit Applied Biosystems (Foster City, CA). ACTB and GAPDH were used as internal controls.

### Immunofluorescence

Adipocytes were characterized using Dye Nile Red (Sigma-Aldrich, St. Louis, MO) and DAPI (Vectashield, Vector, Burlingame, CA) used to according to the manufacturer's instructions. Cells present in the ATM/ASC fractions with and without co-culture were also characterize by immunofluorescence. Cells on the coverslips were fixed with 4% paraformaldehyde/PBS, washed with PBS, treated with 0.2% Triton-X-100, and then blocked with a 4% BSA solution. The samples were incubated with the primary antibodies overnight at 4°C, washed with PBS and incubated for one hour with secondary antibodies labeled with Alexa Fluor 568 and 488 (Invitrogen, Carlsbad, CA). The slides were then washed with PBS and mounted with mounting medium containing DAPI (Vectashield, Vector, Burlingame, CA). Whole tissue mounts were manually cut from excised tissue before being placed in a well and treated identically to cultured cells. Primary antibodies used included: i) DLK (see above); ii) CD14 (see above); iii) CD68, a macrophage, monocyte, and dendritic cell marker (BD Biosciences, San Diego, CA); iv) CD34, an adult hematopoietic stem cell marker (see above) v) CD105, a mesenchymal stem cell marker (BD Biosciences, San Diego, CA). In some experiments preadipocytes were also characterized with S-100 (Sigma Aldrich, St Louis, MO) [27]–[29].

### Labeled nanobead cell lineage analysis

10^7^ cells from ATM/ASC fraction were incubated with 10 µl of a 1∶1 volume slurry of 200–300 nm fluorescent anti-human CD14 nanobeads in PBS (BD Biosciences, San Diego, CA) for 30 minutes at 8°C in 90 µl PBS, 0.5% BSA, 2 mM EDTA, and 0.09% sodium azide. After incubation, the mixture was layered over a Ficoll density gradient as described above to separate any unbound or non-internalized nanobeads from the ATM/ASC fraction, which was then plated for 24 hours and co-cultured as described above.

## Results

### Co-culture of ATMs/ASCs and adipocytes

Isolated adipocytes, prior to co-culture were positive for Nile Red and DAPI staining and were clearly distinguishable from the small amount of free lipids (positive Nile Red and negative DAPI staining) that remained in the adipocyte isolates (**Fig. 1B**). To ensure that adipocytes were not contaminated with ATMs we performed immunostaining in the adipocyte fraction using CD14 antibody, a specific marker for monocytes/macropahges. We were unable to find adipocytes with CD14 (+) ATM cells (**Fig. 1C**). ATMs remained tightly adherent in culture and were negative for Nile Red indicating that these cells did not have any contamination of adipocytes (**Fig. 1D–E**).

### Generation of preadipocytes from the ATM/ASC fraction after co-culture

**Figure 2:**
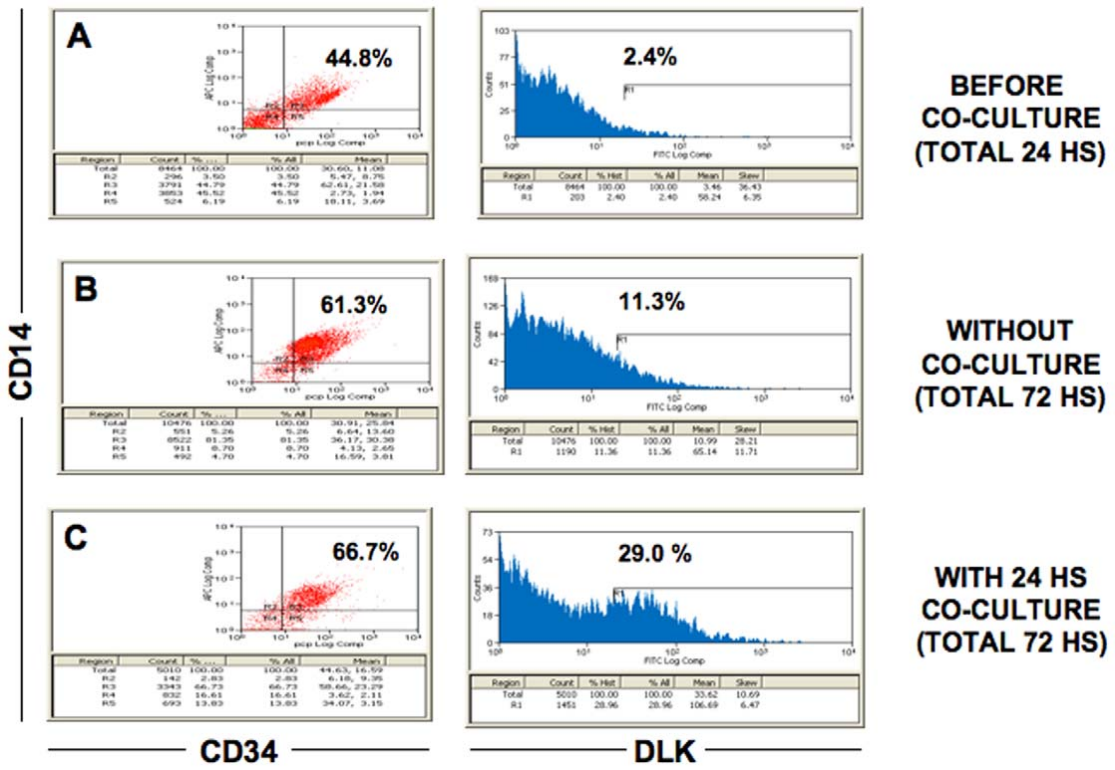
FACS analysis of the ATM/ASC fraction alone and after co-culture with adipocytes. A monoclonal antibody to DLK was used to determine the presence of specific subsets of preadipocytes. (A) Before co-culture (total 24 h), (B) without co-culture (total 72 h), (C) with 24 h co-culture (total 72 h). IgG2a was used as a negative control.

To quantify the number of preadipocytes formed in the ATM/ASC fraction following co-culture, we performed FACS analysis. **Fig. 2** shows the results of a representative experiment of our FACS studies. We used DLK as a marker for preadipocytes [30]–[31], CD14 for ATMs and CD34 for ASCs. We observed that the ATM/ASC fraction, which contains ATMs and ASCs and other cell types, was composed of only 2.4% DLK (+) cells after being allowed 24 hours of growth prior to co-culture **(** **Fig. 2A** **).** The same ATM/ASC fraction maintained for 72 hours of growth without co-culture contained 11.3% of DLK (+) cells, **(** **Fig. 2B**). In contrast, the ATM/ASC fraction that was co-cultured with adipocytes exhibited a significant increase to 29% of DLK (+) cells **(** **Fig. 2C** **).** Results of three different experiments indicate a 3–4 fold increase in DLK (+) preadipocytes in the ATM/ASC fraction after co-culture with adipocytes (p<0.01) (data not shown). Furthermore, adipocytes that were replated after co-culturing and cultured for additional 24 hours demonstrated only minimal additional preadipocyte formation (data not shown). Our results also suggest that preadipocytes could be derived from different population of CD34 (+) and CD14 (+) cells **(** **Fig. 2C** **)**.

### Effect of co-culture in the differentiation of ATM/ASCs to preadipocytes/adipocytes

**Figure 3:**
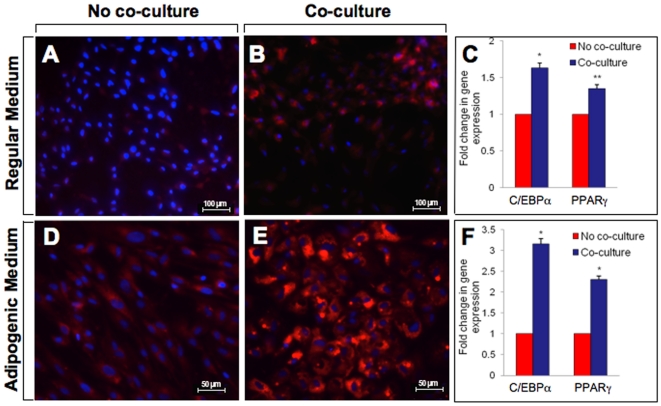
Differentiation of adipose stem cells to preadipocytes/adipocytes. Isolated adipocytes and ATMs/ASCs were cultured separately for 24 hours to allow the cells to reach equilibrium and then were co-cultured for an additional 24 hours. The cells were then separated and ATMs/ASCs were cultured for another 2 days in regular medium followed by an additional 7 days in either the same medium or adipogenic medium (A) ATMs/ASCs without co-culture were cultured in regular medium for 2 days followed by an additional culture of 7 days in the same medium; (B) ASCs/ATMs with co-culture were cultured in regular medium for 2 days followed by an additional culture for 7 days in regular medium. (C) Fold change in gene expression of C/EBPα and PPARγ of ASCs/ATMs with and without co-culture, cell culture conditions are the same as described above (see 3A–B); (D) ASCs/ATMs without co-culture were cultured in regular medium for 2 days followed by an additional culture for 7 days in adipogenic medium; (E) ASCs/ATMs with co-culture were cultured for 2 days in regular medium followed by additional culture for 7 days in adipogenic medium; (F) Fold change in gene expression of C/EBPα and PPARγ of ASCs/ATMs with and without co-culture, cell culture conditions are the same as described above (see 3D–E).

In order to further characterize the preadipocytes formed in the ATM/ASC fraction following co-culture, confluent ATM/ASC fraction without and with co-culture were kept in regular medium (see above) for an additional 7 days. In contrast to the untreated ATM/ASC fraction, preadipocytes derived after co-culture showed many inclusions with lipid droplets (Nile red (+)/DAPI) **(** **Fig. 3A** **vs 3B).** In addition, there were significant increases in C/EBPα (1.6 fold, p<0.01) and PPARγ (1.4 fold, p<0.05) gene expression **(** **Fig. 3C** **)**. To further study the effect of co-culture in adipogenesis, preadipocytes derived after co-culture were kept in adipogenic medium for additional 7 days. Under these conditions, the new preadipocytes contained large lipid drops **(** **Fig. 3D** **vs 3E)**. In addition, there were significant increases in C/EBPα (3.2 fold, p<0.01) and PPARγ (2.6 fold, p<0.001) gene expression **(** **Fig. 3F** **).** These results indicate that co-culture exacerbate adipogenesis at an early stage of differentiation (conversion of ASCs to preadipocytes) as well as during differentiation of preadipocytes to adipocytes.

### Differentiation of ATMs to Preadipocytes

**Figure 4:**
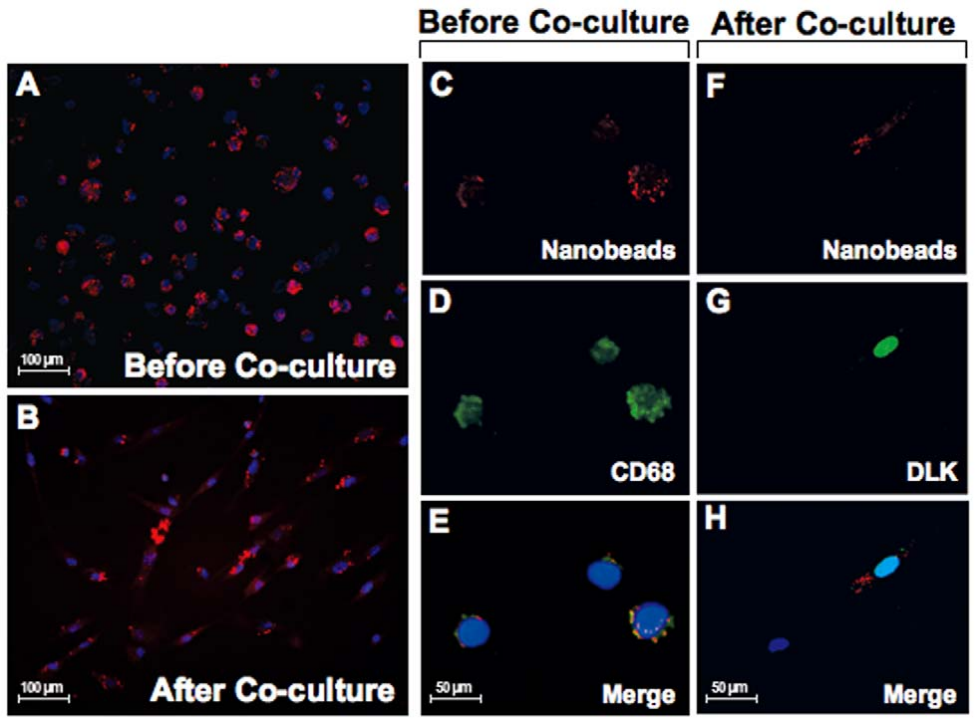
CD14 Nanobead markers before co-culture and after co-culture. Before co-culture. (A) CD14(+) nanobeads in red were incorporated into macrophage fraction; (C) CD14(+) indicates the presence of nanobeads in red, (D) CD68 (+) indicates the presence of ATMs in green; (E) Coincident expression of nanobeads CD14 (+) with CD68(+) ATMs/DAPI. After co-culture: (B) CD14(+) nanobeads in red were incorporated into preadipocytes(F) CD14(+) indicates presence of nanobeads in red (G) DLK (+) indicates presence of preadipocytes in green, (H) Coincident expression of CD14(+) nanobeads with DLK(+) preadipocytes/DAPI.

Preadipocyte formation during co-culture of the ATM/ASC fractions could be attributed to differentiation of CD34 (+) ASCs [12], [14], [32] and other cell components present in the ATM/ASC fraction such as CD68 (+) ATMs. To demonstrate that ATMs could be a source of preadipocytes, we traced ATMs with fluorescent anti-human CD14 nanobeads. We induced ATMs to internalize fluorescence nanobeads prior to co-culture **(** **Fig. 4A, C, D, E** **)**. Indeed, a subset of CD68 (+) ATMs present in the ATM/ASC fraction was induced to phagocytize the nanobeads. Approximately 90% of these CD68 (+) ATMs phagocytized the nanobeads prior to co-culture **(** **Fig. 4A, C, D, E** **)**. After co-culture of the immunofluorescent labeled ATMs/ASCs with adipocytes, a significant number of newly formed preadipocytes contained internalized fluorescent nanobeads suggesting that they were derived from ATMs **(** **Fig. 4F, H** **)**. These nanobead-labeled preadipocytes were also positive for DLK (+) **(** **Fig. 4G, H** **)**, further supporting that the new preadipocytes formed after co-culture were derived from phagocytic cells from CD 68(+) ATMs. There were also examples of DAPI (+)/DLK(-) cells that did not internalize any nanobeads demonstrating the specificity of this assay (**Fig. 4H**).

### Formation of cell spheres after co-culture

**Figure 5:**
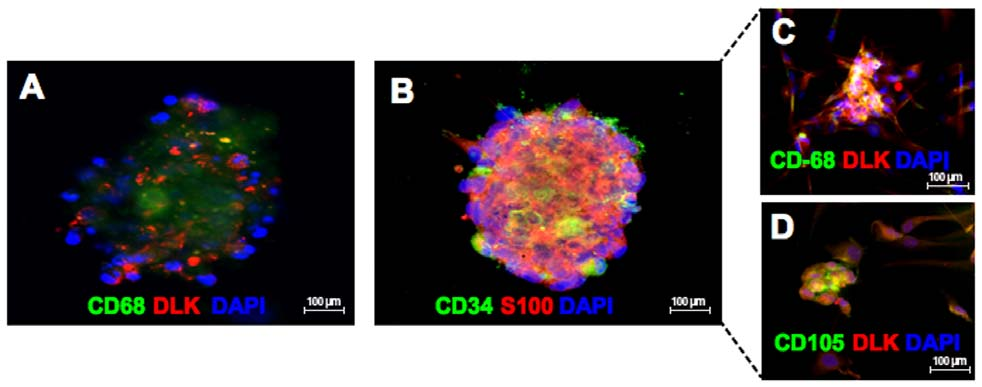
Sphere of human cells formed after 24 hours co-culture between adipocytes, and ATMs/ASPCs. (**A**) Sphere contains CD68 (+)/DLK (+) cells. (**B**) Sphere containing CD34 (+) ASCs/S-100 (+) cells, (**C**) preadipocytes derived from sphere are CD68 (+)/DLK (+) (specific marker for preadipocytes), (**D**) preadipocytes derived from sphere are also CD-105 (+) (marker for mesenchymal stem cells)/DLK (+).

We further characterized the origins of the human preadipocytes formed in the ATM/ASC fraction following co-culture with adipocytes. Unexpectedly, spheres containing CD68 (+)/DLK (+) and CD34 (+)/S100 (+) cells were detected after 24 hours of co-culture, indicating an interaction between preadipocytes and ATMs/ASCs **(** **Fig. 5A, B** **)**. Spheres containing CD34 (+)/S100 (+) cells were also CD34 (+)/DLK (+) cells (Data not shown). From the spheres emerged DLK (+) preadipocytes that also were positive for CD68 **(** **Fig. 5C** **)** and DLK (+) preadipocytes that were positive for CD105 **(** **Fig. 5D** **)**. The finding that the new preadipocytes generated in the ATM/ASC fraction were positive for both DLK and CD105 (classical marker for mesenchymal stem cells) suggests that they originate from mesenchymal stem cell differentiation pathways [11], [12], [14], [32] **.** These results suggest that co-culture between adipocytes with ATMs/ASCs may generate of CD68 (+)/CD34 (+)/preadipocyte cell spheres in an ancillary mechanism to the proliferation of new preadipocytes in humans.

## Discussion

Our results demonstrate that cross-talk between ATMs, ASCs and adipocytes induce the formation of new preadipocytes that can rapidly differentiate into adipocytes. This novel pathway of adipogenesis involves differentiation of ATM to preadipocytes as well as the formation of CD14(+)/CD34(+)/preadipocyte cell spheres from which many preadipocytes are released.

Adipose tissue is a complex population of cells that modulate not only adipose tissue biology, but also insulin sensitivity, reproductive and endocrine systems, immunity, and inflammation [3]. Numerous lines of evidence suggest that inflammation plays a major role in the initiation and maintenance of obesity through adipogenesis. Our present results suggest changes in cell biology resulting from adipocyte interactions with ATMs/ASCs through cell–to-cell contact. Cross-talk between stable adipocyte and macrophage murine cell lines promotes proinflammatory cytokine release [33]. Moreover, interaction between lymphocytes and adipocytes leads to immunoregulation in the body and is responsible for the release of adipokines such as leptin, adiponectin, resistin, and visfatin, as well as cytokines such as TNF-α, IL-6, and MCP-1 [3], [34]. Macrophages block insulin action in adipocytes [9], and in obese individuals macrophages stimulated with proinflammatory cytokines lead to an increase in resistin, an insulin resistance gene. It was recently reported that TNF- α impairs preadipocyte differentiation through the formation of macrophage like preadipocytes [5]. Furthermore, ATMs inhibit the differentiation of human preadipocytes through repression of different transcriptional factors involved in adipogenesis [35]. Recent results indicate that ATMs could play a major role in the production of extracellular matrix proteins that are involved in the cellular transition of preadipocytes to adipocytes [36]. However, the ATM regulation of adipogenesis is complex and has yet to be elucidated. Previous studies suggest that ATMs perform at least two roles in adipose tissue. ATMs phagocytize debris created from rapid adipocyte proliferation, as seen in inflammation associated with obesity. Another function of macrophages is to produce proinflammatory cytokines that increase adipogenesis and insulin resistance [1], [37].

The present study suggests that ATMs also represent a potential source of preadipocytes. In our nanobeads lineage tracing experiments, a significant number of preadipocytes generated after co-culture were found to contain an amount of fluorescent nanobeads comparable to that localized in CD68(+) ATMs prior to co-culture. These results suggest that the CD68(+) ATMs contributed to the formation of new preadipocytes. CD34(+) ASCs are currently considered to be CD14(−)/CD68(−) cells. The number of ASCs present in the ATM/ASC fraction would be insufficient to account for the large number of preadipocytes containing nanobeads in addition to the fact that ASCs would lose nanobeads with each mitotic division, resulting in preadipocytes with few or no nanobeads. Therefore, our results suggest that part of the new preadipocytes originated after co-culture are derived from the ATMs.

Previous studies highlight a complex relationship between lineage and phenotype for macrophages, preadipocytes, and adipocytes. Preadipocytes have been shown to differentiate to macrophages, dedifferentiate from adipocytes, and differentiate back to adipocytes. Macrophages and monocytes can differentiate to CD68 (+), S100 (+), and CD14 (−) dendritic cells, and monocytes in some studies demonstrate characteristics of circulating stem/progenitor cells [38]. Macrophages and adipocytes both express adipsin (complement factor D), the adipocyte differentiation- dependent serine protease gene [39]. Mouse peritoneal macrophages in co-culture with adipocytes can develop long cellular extensions with cytoplasmic lipid vacuoles [9]. These sources of indirect evidence would suggest that plasticity of these cell types does not preclude macrophage to preadipocytes differentiation.

Microarray studies comparing gene expression profiles of macrophages and progenitor/stem cells indicate that both cell types demonstrate the capacity for endocytosis, vesicle trafficking, and actin remodeling [40]. In the same study, preadipocytes were shown to demonstrate phagocytic activity. Approximately 95% of peritoneal macrophages, 45% of mesenchymal stem cells and 35% of preadipocytes were found to display phagocytic behavior, as compared to only 1% of fibroblasts [40]. Phagocytic activity may decline as differentiation proceeds towards more adipocyte-like phenotypes. Thus, phagocytic activity may identify a preadipocyte subset with macrophage progenitors. However, we can not discard the possibility that CD14(+) ATMs and CD34 (+) ASCs could be derived from a common, less differentiated cell type.

**Figure 6:**
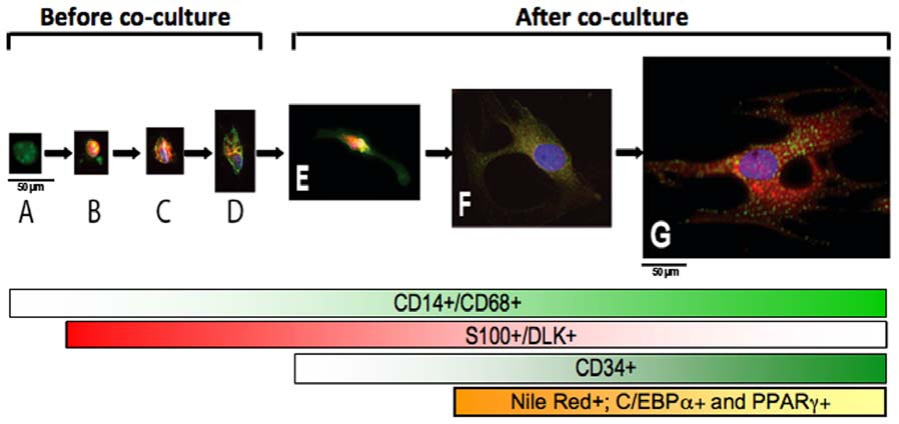
Morphological changes exhibited during CD14 (+) cell differentiation to preadipocytes. Expression of markers CD14 (monocytes/macrophages) (light green), S-100 (preadipocytes/adipocytes) (red), and CD34 (ASCs) (dark green), Nile Red (preadipocytes/adipocytes) (orange). Before co-culture: (A) most of the ATMs are CD14 (+); (B–D) very few ATMs are CD14 (+)/S-100 (+); After co-culture: (E) ATMs become enlarged in size as they transform to preadipocytes (begin to express DLK/S-100 while maintaining CD14 expression); (F) Preadipocytes are S-100 (+) and DLK (+) and start loosing CD14 and CD34 expression; (G) As preadipocytes start differentiating to adipocytes, there is an increase cell size, lipid accumulation (Nile Red (+) cells) and C/EBPα and PPARγ gene expression. The brightness of the color inside the lowers bars indicates the changes in cell expression markers in correlation with the morphological changes exhibited during ATMs differentiation to preadipocytes.

During ATMs differentiation to preadipocytes we observed cells expressing both CD14 (+) and S-100 (+) markers. Most preadipocytes observed were CD14 (+) to varying degrees. As macrophages differentiated towards a preadipocyte morphology, the cells expressed less CD14 and more DLK/S-100 (**Fig. 6**). As CD14 is not expressed by mature adipocytes [41], this suggests that preadipocytes progressively lose CD14 expression or that CD14 cells can dedifferentiate from adipocytes with a concomitant increase of C/EBPα and PPARγ gene expression.

Our FACS data suggest that DLK (+) preadipocytes are derived from both CD14(+) and CD14(−) precursors. Further, within the CD14 (+) group, there exists preadipocytes that are both CD34 (+) and CD34 (−). This would suggest a complex population of cells, with preadipocytes deriving from multiple sources. Macrophages and dendritic cells share the marker CD68, and adipocytes, preadipocytes and dendritic cells are positive for S-100, but only macrophages are CD14 (+) [42]. Similar intermediate differentiation phenotypes have been demonstrated as monocytes or macrophages differentiate to other cell types. Monocytes appear to be able to differentiate into an endothelial precursor population [43], and a chimeric cell that is CD68 (+), S-100 (+), and CD14 (−) has been implicated in diseases such as scleroderma [44].

Interestingly, our co-culture studies demonstrated the formation of CD68 (+)/DLK (+) and CD34 (+)/DLK (+) cells grouped in spheres from which many in preadipocytes were released. Formation of these CD68 (+)/DLK (+) and CD34 (+)/DLK (+) cell spheres could be part of the cellular mechanisms involved in preadipocyte proliferation during co- culture. Recently, unique multipotent cells in adult human mesenchymal populations (multilineage differentiating stress enduring cells, “Muse cells”) have been isolated from human skin fibroblasts or bone marrow stromal cells [45]. These cells are characteristically known to form spheres [45]. The CD68(+)/CD34(+) cell spheres originated after co-culture (**Fig. 5A, B**) from which preadipocytes derived, could also have similar pluripotent characteristics as Muse cells.

*In vitro* expanded, autologous preadipocytes have the potential for future therapeutic applications, since adipocytes, ATMs and ASCs are an abundant and easily harvested cell type. The results of our co-culture experiments demonstrate that surgically-derived adipose tissue can serve as a source of cells that, through co-culture, can be used for enhanced production of preadipocytes. Preadipocyte proliferation and differentiation does not exclusively lead to adipogenesis. In turn, preadipocytes can be directed to differentiate down osteogenic, chondrogenic, adipogenic, myogenic, cardiomyogenic, neurogenic, angiogenic, or dendritic, as well as adipogenic, pathways. Thus, therapeutic modulation of preadipocyte differentiation may provide new insights that may lead to novel treatments for tissue regeneration and reconstructive medicine.

Furthermore, understanding the regulation of the conversion of ATMs to preadipocytes, and preadipocyte proliferation could offer a new way to influence adipogenesis, leading to new treatments for obesity, inflammation and type 2 diabetes.
